# Italian healthcare professionals' real-world experience with immunization in children of non-European origin: the Vax4globe survey

**DOI:** 10.3389/fped.2026.1774399

**Published:** 2026-06-09

**Authors:** Viviana Moschese, Simona Graziani, Antonietta Spadea, Maurizia D’Amore, Raffaella Mosco, Sara Ciampini, Nicola Di Giorgio, Susanna Arcano, Simona Ceccarelli, Marco Chianca, Simona Piccinini, Antonella Polito, Marta Porcari, Pamela Puliafito, Romina Silenzi, Elisabetta Del Duca, Alfonso Piciocchi, Claudio Pignata, Michele Miraglia Del Giudice, Gian Luigi Marseglia, the Italian Society of Pediatric Research (SIRP) and the Italian Society of Pediatric Allergy and Immunology (SIAIP) Vaccine Committee

**Affiliations:** 1 Pediatric Immunopathology and Allergology Unit, Policlinico Tor Vergata, University of Rome Tor Vergata, Rome, Italy; 2 Local Health Agency RM2, Rome, Italy; 3 Vaccination Complex Operational Unit of Local Health Agency Rome 1, Rome, Italy; 4 UOC District Management 6.6, Local Health Agency Rome 6, Anzio, Italy; 5 Local Health Agency RM5, Rome, Italy; 6 Local Health Agency RM1, Rome, Italy; 7 Local Health Agency RM6, Rome, Italy; 8 GIMEMA Foundation, Rome, Italy; 9 Department of Translational Medical Science, Federico II University, Naples, Italy; 10 Department of Woman, Child and General and Specialized Surgery, University of Campania “Luigi Vanvitelli”, Naples, Italy; 11 Department of Clinical, Surgical, Diagnostic and Pediatric Sciences, University of Pavia, Pavia, Italy

**Keywords:** childhood, public health, vaccination, vaccine equity, vulnerable populations

## Abstract

**Introduction:**

Nowadays vaccine hesitancy represents a significant current global health threat, causing reduced vaccine coverage with relevant effects on vulnerable populations. This study explores Italian healthcare professionals' (HCPs) experience with immunization of non-European (non-EU) origin children to implement targeted strategies for higher vaccine coverage and a “vulnerables-friendly care”.

**Methods:**

The Vax4globe survey was administered to HCPs distributed throughout the Lazio Region from February to July 2024. The survey included HCPs' demographic data and twelve items investigating vaccine compliance and barriers of non-EU origin children in their clinical practice as well as potential strategies to increase immunization.

**Results:**

The survey included 101 HCPs, namely physicians and pediatricians working at vaccination centers and primary care services, caring for approximately 50,000–60,000 children overall, of which approximately 15,000 were of non–EU origin. As stated by HCPs, children's country of origin were mainly from countries in Asia (median 30%; range 0%–80%) and South America (median 20%; range 0%–30%). Of the HCPs, 88% (95% CI: 80.4–93.1) reported that ≤50% of non-EU origin parents were able to provide evidence of their children's immunizations and, according to 80% (95% CI: 71.4–86.8) of HCPs, the Italian National Vaccination Prevention Plan (PNPV) was appropriate in ≤50% of children at first assessment. Therefore, according to HCPs, the vaccination record of the country of origin is essential to verify the immunization status (57%), PNPV vaccines should be recommended according to patient's age and documented immunization status (62%), and priority should be given to mandatory vaccines in children under 7 years of age not compliant with the PNPV (96%). In our survey, adherence to PNPV vaccines was significantly higher than to Sars-CoV-2 vaccine in pediatric age patients (immunization adherence <10%: 3% PNPV vs. 48% Sars-CoV-2; immunization adherence >90%: 42.5% PNPV vs. 0% Sars-CoV-2) (*p* < 0.0001). Similarly, the adherence to Sars-CoV-2 vaccine was lower in children than in their parents (immunization adherence <10%: 48% vs. 17%, respectively) (*p* < 0.0001). Finally, according to HCPs, the main barriers to vaccine adherence were linguistic and socio-cultural (75%;95% CI: 66.0–82.6) for PNPV vaccines and safety concerns (52%; 95% CI: 42.8–61.9) for the Sars-CoV-2 vaccine.

**Discussion:**

Our study reports a real-world picture of immunization among non-EU origin children by Italian HCPs and highlights the need for targeted strategies to overcome factors affecting vaccine hesitancy. To date, vaccine health equity is far from being achieved.

## 1 Introduction

Vaccinations are a cornerstone of public health due to their role in infant survival (1). Nevertheless, in 2024, children who had not received any vaccinations, defined as zero-dose children, and partially vaccinated children amounted to approximately 14.3 million and 5.6 million, respectively (2). In recent years, escalating vaccine hesitancy worldwide along with reduced vaccine coverage and increased risk of vaccine-preventable disease outbreaks have caused devastating effects on vulnerable people. Therefore, the World Health Organization (WHO) has included vaccine hesitancy among the ten main threats to global health (3).

Vaccine hesitancy arises from an imbalance between the awareness of immunization benefits and a misperception of adverse effects. It is a complex phenomenon affected by several economic, socio-cultural, and individual factors. Indeed, as reported by the Strategic Advisory Group of Experts Working Group (SAGE WG), determinants of vaccine hesitancy are heterogeneous and may be influenced by time, place, and type of vaccine (4). Particularly, according to the SAGE WG, factors associated with vaccine hesitancy belong to three key domains: contextual influences including historic, socio-cultural, environmental, health system/institutional, economic, and political factors; individual and group influences related to personal perception of the vaccine or influences of the social environment; and vaccine and vaccination-specific issues related to the type of vaccine or vaccination process (4). Migrant children or children with parents of foreign origin may experience social marginalization, limited healthcare access, and poor socioeconomic status, reflecting their vulnerability and declining in vaccine confidence (5–8). In this context, as reported by the WHO Measuring Behavioural and Social Drivers of Vaccination group (9), healthcare professionals (HCPs) play a pivotal vaccine referral role for parents (10). To ensure higher immunization compliance, it is essential for HCPs to both be adequately updated and trained on vaccines and to know and use clear and comprehensive communication strategies that take into account the social background of each single foreign family (11). Indeed, recent studies have demonstrated that immunization coverage is positively related to the importance given by parents to the information provided by HCPs (12) and that the attitude and sensitivity of HCPs affect parents' decision to adhere to vaccination (13). Also, previous studies have highlighted the importance of health science degree programs to provide students with extensive information on vaccines and on the severity of vaccine-preventable disease to support vaccination campaigns (14, 15).

Overcoming vaccine hesitancy is crucial to achieving the objectives of the European Immunization Agenda 2030, which aims to ensure equitable access to vaccines, promote vaccination at all stages of life, and implement locally tailored solutions to increase vaccination uptake in the population. These efforts contribute to advancing universal health coverage and sustainable development (16). Furthermore, fifty years ago the WHO launched the Expanded Programme on Immunization to ensure universal access to relevant vaccines to achieve disease control and enhance health outcomes in tandem with other public health programs (17).

In Italy, there are 1.3 million children born to foreign parents, also called second-generation migrants, of whom about 1 million hold non-Italian citizenship, corresponding to approximately 11.2% of residents between 0 and 17 years old in 2021 (18). As previously reported, migrant families frequently experience difficulties in accessing and interacting with health services (19, 20). Our study, carried out in the framework of the Italian Society of Pediatric Research (SIRP) and the Italian Society of Pediatric Allergy and Immunology (SIAIP), aims to investigate vaccination adherence among families of non-European (non-EU) origin for their children, as well as reasons for vaccine refusal/delay as observed by HCPs in their clinical practice. The study aims to contribute to strategies to improve vaccine adherence in non-EU origin children and reduce inequalities for a “vulnerables-friendly care” approach.

## 2 Materials and methods

### 2.1 Study design and study population

The Italian Society of Pediatric Research (SIRP) and the Italian Society of Pediatric Allergy and Immunology (SIAIP), in collaboration with the Pediatric Immunopathology and Allergology Unit of Tor Vergata University Hospital, prepared this survey to investigate the knowledge and attitude of non-EU origin parents on pediatric immunization as to the Italian National Vaccination Prevention Plan (PNPV) according to HCPs’ experience. The study was carried out over a 6-month period and the questionnaire was filled out by HCPs from vaccination centers and primary care services variously distributed in different areas of Rome and its provincial districts.

### 2.2 Data collection

A survey form was created for this cross-sectional study according to the specific literature (21). The anonymous questionnaire was available in paper format and on the Google Forms electronic platform. The first questionnaire section collected the HCPs' demographic and employment profile, while the second section included twelve items on the HCPs’ experience regarding vaccine compliance of non-UE origin children, the main barriers for non-EU origin parents to comply with the Italian PNPV schedule for their children, the Sars-CoV-2 vaccine adherence among non-EU origin families with the main reasons for vaccine refusal, and the most appropriate strategies to improve immunization rates.

### 2.3 Data analysis

Participants' characteristics for the whole population were summarized by means of frequencies and percentages. To identify significant factors associated with vaccine adherence and barriers, Chi-Square or Fisher's exact test were used. All tests were two-sided with a significance level of 0.05, and all the analyses were performed using the R software (R Core Team. 2023).

### 2.4 Ethical considerations

Following explanation of the main objectives of the study, informed consent was verbally secured from all participating HCPs. Since the questionnaire was anonymous without personal identifying information, no Ethics Committees approval was necessary to lead the survey.

## 3 Results

### 3.1 HCPs' general features and their experience with immunization of non-EU origin children

In total, 101 HCPs completed the questionnaire, of whom 91 (90%) were women. Most of the participants were aged between 40 and 60 years (54%), followed by those aged 30–40 years (26%) and >60 years (20%).

Participating HCPs, namely physicians and pediatricians working at vaccination centers and primary care services, cared for an estimated total of 50,000–60,000 children annually, including approximately 15,000 children of non-EU origin, providing a broader real-world view of immunization dynamics among non-EU populations. HCPs reported that the children were mainly of Asian (median 30%; range 0%–80%) or South American (median 20%; range 0%–30%) descent, with fewer children from African (median 11%; range 0%–95%) or European countries not belonging to the European Union (median 10%; range 0%–50%).

Investigating the HCPs' experience on the immunization status of non-EU origin children, we observed that, according to 88% (95% CI 80.4–93.1) of HCPs, the percentage of non-EU origin parents able to provide evidence of their children's vaccination record was ≤50% (Table 1). Furthermore, 80% (95% CI 71.4–86.8) of HCPs believe that the PNPV was matched in ≤50% of non-EU children at first assessment (Table 1).

**Table 1:** HCPs’ experience with the immunization status of non-EU origin children.

Immunization adherence categories (%)	HCPs *n* = 101 (%)	95%CI
Parents able to prove their children's immunization status (%)		
<10%	51 (50%)	40.9–60.0
10–50%	38 (38%)	28.8–47.4
50–90%	11 (11%)	6.2–18.5
>90%	1 (1%)	0.2–5.4
Children with immunization status appropriate to PNPV at first assessment (%)		
<10%	31 (31%)	22.5–40.3
10–50%	50 (49%)	40.0–59.1
50–90%	18 (18%)	11.6–26.4
>90%	2 (2%)	0.5–6.9

As reported in Table 2, due to the relevance of the immunization status for vaccine-preventable diseases in pediatric age, 57% of HCPs believe that the vaccination record of the country of origin is important, together with direct information request to the parent/caregiver for 42% of HCPs. Furthermore, 62% of HCPs affirm that vaccine recommendations according to PNPV should be provided considering the patient's age and documented immunization status. Conversely, 23% of HCPs believe that an immunization schedule should be offered regardless of age and knowledge of previous vaccinations. Moreover, 96% of HCPs believe that mandatory vaccines for school attendance represent a priority in children <7 years of age who did not match the PNPV. As for Hepatitis A (HAV) vaccine, 69% of HCPs believe that, both in case of planned travel to endemic countries or hosting relatives from an endemic country of origin, any child should be immunized regardless of clinical history and serological tests, whereas 31% of HCPs state that this vaccine should be carried out only in children with a negative clinical history and serology. Most HCPs (95%) agree that a combined strategy is required to improve vaccine uptake of non-EU origin children, which comprises vaccine inquiries at child's first assessment, the development of dedicated national immunization guidelines for HCPs, and awareness campaigns.

**Table 2:** HCPs’ responses to the survey on the immunization of non-EU origin children.

Question	Response Options	*N* = 101 (%)	95%CI
How should immunization status for vaccine-preventable diseases be checked? (1 unknown)	Vaccination record of origin country	58 (57%)	48.2–67.2
	Direct request to the parent/caregiver	0 (0%)	0.0–3.7
	Vaccination record of origin country and direct request to the parent/caregiver	42 (42%)	32.8–51.8
How should an immunization schedule be proposed according to the PNPV? (2 unknown)	To everyone, regardless of age and immunization status	23 (23%)	16.0–32.5
	To everyone, only for undocumented/uncertain vaccines and regardless of the patient's age	8 (8%)	4.2–15.1
	According to the PNPV based on the patient's age and certified immunization status	63 (62%)	53.8–72.4
	According to the PNPV based on the patient's age and regardless of certified immunization status	5 (5%)	2.2–11.3
Which vaccines should be prioritized in non-EU origin children <7 years who don’t comply with PNPV?	Anti-pneumococcus and MenB	1 (1%)	0.2–5.4
	MMR	0 (0%)	0.0–3.7
	MMR and MenB	3 (3%)	1.0–8.4
	Priority for mandatory vaccines to attend school	97 (96%)	90.3–98.4
To which patient would you like to/could you recommend the Hepatitis A vaccine?	No one, under any circumstances	0 (0%)	0.0–3.7
	Only if the parent/caregiver reports a negative medical history and/or negative serology, in case of travel to endemic countries	31 (31%)	22.5–40.3
	To everyone, without performing serology and regardless of medical history in case of travel to endemic countries	70 (69%)	59.7–77.5
Which is the most effective method to improve immunization coverage in non-EU origin children? (1 unknown)	Survey among parents at first assessment	0 (0%)	0.0–3.7
	Development of national guidelines for this specific population addressed to HCPs	1 (1%)	0.2–5.4
	Awareness campaign carried out by the health authority addressed to this vulnerable population	3 (3%)	1.0–8.5
	All of the above	96 (95%)	90.2–98.4

### 3.2 Children's immunization compliance according to PNPV by non-EU origin parents

**Figure 1:**
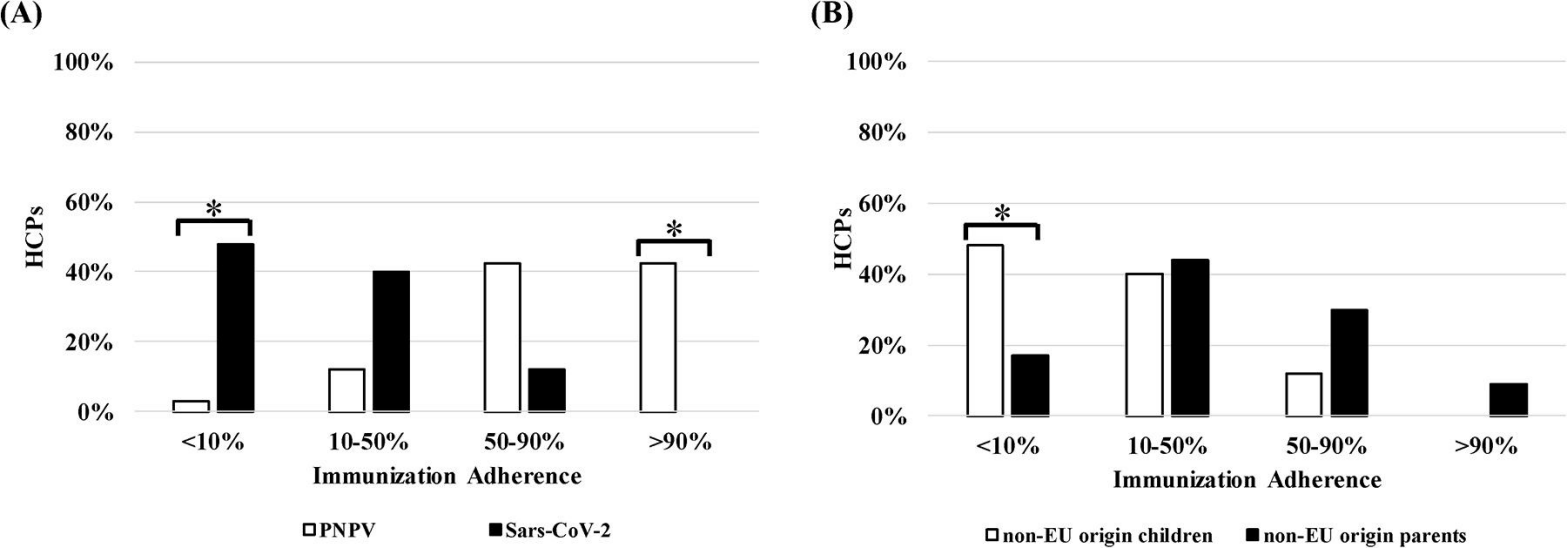
Immunization adherence according to HCPs: **(A)** PNPV vaccines vs. Sars-CoV-2 vaccine in non-EU origin children; **(B)** Sars-CoV-2 vaccine in non-EU origin children vs. their parents. Symbols for *p*-values: *<0.0001.

**Figure 2:**
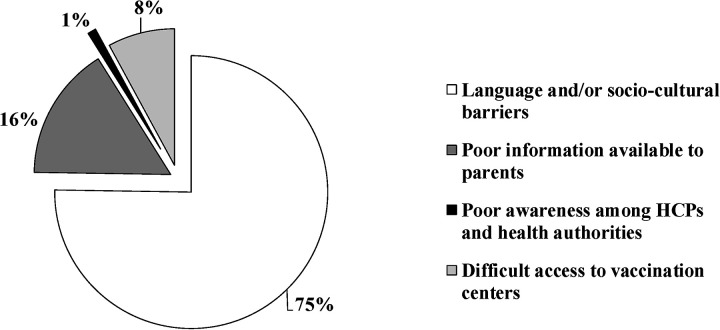
Main barriers for non-EU origin parents to comply with PNPV vaccines for their children according to HCPs.

When the vaccination adherence of non-EU origin children as to the PNPV was investigated, 43% of HCPs reported a frequency of adherence matching the PNPV immunization coverage target of >90% (Figure 1A). Particularly, HCPs reported linguistic and socio-cultural barriers (75%; 95% CI: 66.0–82.6), lack of vaccine information (16%), and difficulty in accessing vaccination centers (8%) as the main hindrances for non-EU origin parents (Figure 2).

### 3.3 Immunization compliance for Sars-CoV-2 vaccine in non-EU origin families

When the Sars-CoV-2 vaccination adherence of non-EU origin children was investigated, none of HCPs reported an immunization coverage >90%, a frequency of adherence in stark contrast to the previously mentioned 43% for PNPV vaccines (*p* < 0.0001) (Figure 1A). In line with a much lower pediatric Sars-CoV-2 vaccination coverage, 48% of HCPs reported a < 10% frequency of Sars-CoV-2 immunization vs. 3% when PNPV vaccines were considered (*p* < 0.0001) (Figure 1A). Of note, according to HCPs, the frequency of Sars-CoV-2 immunization adherence is strikingly different between parents and children. Indeed, the pediatric immunization adherence was predominantly <10% and between 10%–50% according to 48% and 40% of HCPs, respectively. Conversely, the parents' immunization adherence was predominantly between 10%–50% and 50%–90% according to 44% and 30% of HCPs, respectively. Again, the frequency of immunization <10% for Sars-CoV-2 vaccine was significantly higher in children (48%) compared to their parents (17%) (*p* < 0.0001) (Figure 1B).

Finally, according to HCPs, the main reasons for Sars-CoV-2 vaccine hesitancy of non-EU origin parents are mainly due to safety (52%; 95% CI: 42.8–61.9) and need concerns (39%; 95% CI 29.7–48.4), low information received (33%), and non-mandatory vaccine status (30%) (Table 3). On the other hand, ethical-religious reasons and unease in booking vaccinations are rarely reported.

**Table 3:** HCPs’ reports on Sars-CoV-2 vaccine hesitancy in parents and children of non-EU origin.

Reported reasons for Sars-CoV-2 vaccine hesitancy	*N* = 101 (%)	95%CI
Doubts about vaccine safety	53 (52%)	42.8–61.9
Doubts about the need for vaccination	39 (39%)	29.7–48.4
No information received about vaccine	33 (33%)	24.3–42.3
Non-mandatory vaccine status	30 (30%)	21.7–39.2
Doubts about vaccine efficacy	25 (25%)	17.4–34.0
Preference for “natural immunity”	10 (10%)	5.5–17.3
Ethical-religious reasons	7 (7%)	3.4–13.6
Difficulty to book an appointment at the Vaccination Center	6 (6%)	2.8–12.4

## 4 Discussion

Over time, immunization has proven to be a pivotal tool for the prevention of transmissible diseases. This role is becoming even more relevant due to a growing trend of vaccine hesitancy, which represents a public health challenge, which is experienced most significantly by marginalized, disadvantaged, and vulnerable populations where low vaccination rate has been widely demonstrated (22, 23). In this context, HCPs play a key role, since they are on the front line for vaccine outreach. Our study aims to evaluate HCPs' notions on the immunization status of non-EU origin children and on the main barriers to vaccination. This is done with the aim to improve vaccine strategies and increase vaccination uptake and equity in vulnerable populations.

Italy currently hosts a large and heterogeneous foreign resident population, and recent migration flows remain substantial, making the management of preventive care in migrant children an increasingly relevant public health issue. As of 1 January 2025, there were 5.422 million foreign residents in Italy, corresponding to 9.2% of the total resident population (24). This data is constantly evolving since, between 2023 and 2024, an immigrant average increase of 6.4% compared to 2022 was observed, driven exclusively by the positive variation in the government admissions of foreign citizens. The most significant increases concern flows from Africa (+43.9%), followed by America and Oceania (18.5% overall) and Asia (+12.5%) (25). However, HCPs reported that the non-EU children annually assessed come predominantly from Asia and South America. The difference between the Italian national data and what was reported by our HCPs may be due to the variable distribution of foreign communities not only among Italian regions but also within neighborhoods of the same city. Furthermore, these data may be affected by foreign citizens' access to healthcare services, which may be impeded by document irregularities due to uncontrolled entry, expiration of residence permits, and rejection of regularization requests.

According to 80% of HCPs, less than 50% of non-EU origin children matched the PNPV vaccine schedule at the time of first assessment. Our data agree with other studies reporting the high inequality in immunization rate between native children and children of foreign origin (26, 27). Indeed, a lower vaccination coverage in children of Black, African, and Caribbean origin compared to white British children has been reported (28). Estimates of current global vaccination coverage have highlighted that most zero-dose children come from conflict-affected regions and those with limited resources available for immunization, such as sub-Saharan Africa. As of 2023, Nigeria, India, the Democratic Republic of Congo, Ethiopia, Somalia, Sudan, Indonesia, and Brazil host more than 50% of the 15.7 million global zero-dose children (29). Therefore, these inequities may affect non-EU families’ attitudes and knowledge toward vaccination and highlight the need to reinforce vaccine campaigns for these vulnerable populations.

As to 88% of Italian HCPs, parents of foreign origin are frequently unable to document the immunization status of their children. This might be ascribed to the phenomenon of migration, different vaccination schedules among countries, availability of immunization documentation, differences in catch-up strategies, different ways of accessing vaccination centers, and distinct relevance given to immunization by various ethnic groups. In Italy, PNPV 2023–2025 represents the national regulatory framework developed to harmonize the vaccination strategies through age-based recommendations and specific catch-up doses guidelines, thus guaranteeing the population the full benefits of vaccination, regardless of place of residence, income, socio-cultural level, or legal status (30). To verify the immunization status, HCPs require support of vaccination records from the country of origin (57%) combined with direct request to parents (42%). When the vaccination series is not completed, the PNPV schedule according to the child's age and precedence for mandatory vaccines to attend school are to be prioritized by 62% and 96% of HCPs, respectively. This is essential to ensure adequate vaccination coverage, as also established by Italian PNPV, and therefore avoid outbreaks of vaccine-preventable diseases, as previously occurred with measles in several European countries (31). Furthermore, the development of a digital vaccination certificate that can be updated, electronically stored, easily accessible, and provide proof of a person's immunization status is increasingly emerging, thus highlighting how innovative technologies may be pivotal in the healthcare system (32). Therefore, favoring a safe and effective vaccine offer that also responds to the principles of equity, homogeneity, and accessibility is crucial to maintain a high global vaccine coverage.

Since international travel is increasing and may be more frequent among people who reside outside of their country of origin, it should become common clinical practice to inquire about travel plans abroad and provide detailed vaccination information based on the travel destination. Notably, the Centers for Disease Control and Prevention (CDC) has a list of vaccines required for travel, including the HAV vaccine, since this disease is still endemic in areas such as Africa, Asia, the Middle East, Central and South America, and Eastern Europe and has an incidence of 6.0–30.0 cases per 100,000 person-months in non-immune travelers to at-risk destinations (33). Our survey showed that, according to 69% of HCPs, both in case of planned travel to endemic countries or hosting relatives from an endemic country of origin, any child should be immunized regardless of clinical history and serological tests. This is a hot issue that should be reinforced among our HCPs to draw greater attention toward those infectious diseases that are less frequent in our country but that can re-emerge given the high frequency of travel abroad. Indeed, a recent French study showed that a very small percentage of child travelers in highly endemic areas was vaccinated, highlighting the need to improve the population's knowledge, including that of HCPs (34). Furthermore, in their study, Herrera-Restrepo et al. showed that even if HCPs are aware of the HAV vaccine's importance, vaccination rates are still low, suggesting the need to strictly follow vaccine recommendations (35). Therefore, vaccination updates and, in case of symptoms, investigation into family members arriving from abroad could become two key inquiries during HCPs' daily clinical practice in an increasingly multi-ethnic society.

When pediatric Sars-CoV-2 vaccination adherence was analyzed, there was a significantly lower uptake compared to non-EU origin parents (immunization adherence <10%: 48% vs. 17%) (*p* < 0.0001) which was even more marked when compared to PNPV vaccines (immunization adherence <10%: 48% vs. 3%) (*p* < 0.0001). This is in agreement with previous studies that report low vaccination rates for Sars-CoV-2 in the pediatric age compared both to adults and PNPV vaccines (36–38).

The difference between adults and children in SARS-CoV-2 vaccination adherence may be affected by various factors, such as mandatory vaccination requirements for adults to access various settings including workplaces, the perception of low infection severity in the pediatric age, and the non-compulsory requirement of this vaccine for children. As to the higher adherence to PNPV than to Sars-CoV-2 vaccines, this may be fostered by the frequent growth follow-ups in the first year of life when pediatricians can provide more information on vaccines and their requirement for entrance to school. Language and socio-cultural barriers are reported as the main reasons that affect PNPV vaccine compliance whereas doubts on safety, need for vaccination, and lack of information favor Sars-CoV-2 vaccine hesitancy. A recent study highlighted those factors associated with Sars-CoV-2 vaccine acceptance and hesitancy worldwide and a higher vaccine acceptance in Europe and Asian countries than American (39). Furthermore, the status of migrants with both parents born abroad has been identified as a main risk factor for lack of a Sars-CoV-2 vaccination (40). A clear understanding of those factors that directly or indirectly impact on the immunization of non-EU origin children is pivotal to support vaccination campaigns for vulnerable people. An Italian survey on HPV immunization campaigns and strategies reported that organizational aspects such as access to vaccine centers, vaccine recall systems, and the use of a regional immunization registry are among the major contributors to vaccine coverage (41). Also, as reported in a French study, in-school combined vaccine-related education and vaccination campaigns can improve immunization uptake (42). All this is of particular relevance to support parents of foreign origin who face administrative and linguistic challenges. In this context, HCPs' experiences are extremely relevant as a trusted source for vaccine-related information, and immunization at their office is usually preferred (43). Therefore, HCPs' training is essential to track compliance with the Italian PNPV and any other additional vaccines and to provide adequate information on the risks of vaccine-preventable infectious diseases. A recent Japanese study highlighted medical students' awareness of the need to improve their immunization knowledge, focusing on hesitancy and communication skills to favor immunization uptake (44). Also, the need to improve the knowledge of nurses and pharmacists has also been outlined (44). HCPs' vaccine knowledge and confidence have been proven to be closely related to vaccination uptake rates (45), and targeted and clear communication on immunization topics between HCPs and patients promotes vaccination adherence (46). Appropriate patient-centered communication strategies are to be deployed for a trust-based doctor-patient relationship and informed-decision making, including vaccine risks and benefits to obtain positive immunization adherence outcome (47). Recently, Skirrow et al. reported that the lack of adequate time for healthcare workers to provide correct information and the absence of a linguistic/cultural mediator often led to lower vaccination adherence by parents of foreign origin compared to natives (48). Therefore, time and training are pivotal for healthcare workers who need to deal with non-EU parents by providing clear and simple information with no pressure but with empathy and shared decision-making. The creation of distinct vaccination campaigns for vulnerable people, such as families of foreign origin, in selected places of daily life (e.g., schools, churches, receptions, and social centers) may favor immunization through personalized plans and healthcare. A community-based participatory research program involving multidisciplinary collaboration among researchers, local physicians, and community members, as the one implemented for Sars-CoV-2 in California (49) or in Brazil (50), might represent a model to reduce health inequities and vaccine hesitancy. Therefore, to improve accessibility and confidence in vaccination programs among non-EU origin parents, it is pivotal to implement strategies on multiple levels of evidence-based intervention (communication, healthcare personnel training, and organization of healthcare facilities), with flexibility and dynamism in order to adapt to continuous environmental and social changes (41, 51–53).

The success of the respiratory syncytial virus (RSV) vaccine, recently approved in Italy for all newborns and administered at birth centers, underlines the importance of pediatric immunization with no access barriers and bureaucracy to ensure the adequate protection of children. Thus, scheduled booster doses or simultaneous vaccine administrations with cultural and linguistic appropriate health information and easy access to health services together with appropriate use of social media may be targeted interventions to ameliorate vaccine uptake and reduce the risk or reemergence of vaccine-preventable diseases with lesser hospitalization rates for vulnerable people (54–57).

In brief, the development of a comprehensive training program on immunization with correct communicative skills involving all categories of HCPs and community-specific campaigns are pivotal to reaching socio-culturally vulnerable populations. Moreover, promising results are also being observed from the use of artificial intelligence (58, 59) which, through its methods, could aid healthcare work to safeguard vaccine safety, quality and equity.

### 4.1 Limitations

Our study has several limitations to be considered. First, the study involved a limited sample of HCPs from a small geographic area, thus preventing generalization across society. Second, since the majority of participating HCPs were pediatricians or operators of vaccination centers, they were more likely to vaccinate and provide information to parents and therefore their attitudes and perceptions may not represent the general views of the whole healthcare community. Third, since all collected data were self-reported by participating HCPs, they may be subject to a higher risk of information bias. Finally, as the questionnaire did not collect data on the specific countries of origin of the children/families, preventing a more granular interpretation of the results based on differences in immunization schedules, documentation systems, and vaccine policies across countries.

## 5 Conclusions

Our study provides the views and experiences of Italian HCPs in their clinical practice on the immunization of non-EU origin children. Our findings highlight the need to better understand misconceptions, perceptions, and barriers to vaccination adherence of non-EU origin parents for their children. Overall, according to HCPs, the implementation of targeted strategies, such as the development of specific guidelines for HCPs to address vaccine hesitancy and appropriate training on effective communication skills, is essential to create a relationship between healthcare professionals and patients that respects the uniqueness of the patient with their own religious and cultural beliefs. By overcoming vaccine hesitancy and differences in vaccination policies across countries, we will be able to strengthen the pivotal role of immunization within the framework of the international human right to health and provide more vulnerable-friendly healthcare.

## Data Availability

The raw data supporting the conclusions of this article will be made available by the authors, without undue reservation.
